# Editorial: Hopelessness and suicide among children and adolescents in low and middle income countries

**DOI:** 10.3389/fsoc.2023.1215073

**Published:** 2023-06-01

**Authors:** Godfrey Zari Rukundo, Raphael Emeka Ogbolu, James Mugisha

**Affiliations:** ^1^Department of Psychiatry, Mbarara University of Science and Technology, Mbarara, Uganda; ^2^African Centre for Suicide Prevention and Research, Mbarara, Uganda; ^3^Suicide Research and Prevention Initiative (SURPIN), Lagos, Nigeria; ^4^Faculty of Social Sciences, Kyambogo University, Kampala, Uganda

**Keywords:** suicide, hopelessness, suicidal attempt, suicidal ideation, low and middle income countries

## Background

*Although s*uicide is a potentially preventable condition, it has remained global leading cause of death. The factors associated with death by suicide are similar to those identified in persons with suicidal ideation and attempts (Oppong Asante et al., 2021; Quarshie et al., 2021; Liu et al., 2023; Zhao et al., 2023). For effective formulation of preventive strategies to be put in place, there is need to first understand the modifiable risk factors and the possible protective factors that need to be enhanced. Studies conducted in one geographical region cannot necessarily apply in another (Zheng et al., 2023). This editorial summarizes data from recent studies among children and adolescents in low and middle income counties (LMICs).

A study by Bukuluki et al. among 219 vulnerable adolescents in Uganda reported a 46.1% prevalence of suicidal attempt. Given the high association between suicidal attempt and eventual death by suicide, this prevalence is quite alarming. In the same study, suicidal ideation was reported by 30.6% in the past 4 weeks preceding the survey. Sadly, over 75% of the youth with suicidal plans in the week preceding the survey had a clear suicide plan that could easily be executed. The most common factors associated with suicide in this study are preventable and included sexual relationship-related challenges, family breakdowns, domestic violence, trauma and abuse alcohol or drugs Bukuluki et al. Suicidal ideation was lower among adolescents who knew where to obtain professional psychological care. Unfortunately, there are fewer places that offer accessible adolescent friendly or sensitive services.

Another study by Quarshie in urban Ghana reported a prevalence of 38.7% for self-harm behavior and 45.8% self-harm ideation over a period of 12-months. The author reported that self-harm ideation and behavior were associated with bullying, physical abuse, conflict with parents and alcohol use (Quarshie). The study by Namuli et al. in Uganda assessed the prevalence and factors associated with suicidal ideation among 271 HIV infected children and adolescents. Suicidal ideation was associated with higher depressive score, somatic symptoms, thought problems, social problems and rule breaking behavior (Namuli et al.). Children and adolescents who obtained higher total scores on the parent child relationship scale (PCRS) were associated with lower reports of suicidal ideation.

Similarly, a study by Bao et al. reported that being bullied more frequently could led to higher rates of suicide attempts. The overall prevalence of suicidal attempt was 25% among people who were bullied about their religion. The other factors associated with suicidal attempt were sleep deprivation, being kicked, pushed, or shoved, having high-BMI and being left out of activities (Bao et al.). Similar to the study by Namuli et al. in Uganda, the study by Zheng et al. in China reported that there the mother-child relationship had direct impact on suicidal ideation. In addition, Zheng et al. also reported that there was association between suicidal ideation and father-child relationship, teacher-student interaction and peer-peer relationships.

## Summary

Suicide is a major concern among young people in low and middle income countries. From the foregoing, suicidality among children and adolescents is influenced by many factors among which bullying and parental relationship seem to stand out. This is not surprising considering the developmental stages that children and adolescents belong to and the associated challenges. An accurate understanding of the risk and protective factors of suicide in this setting is essential before effective and pragmatic prevention and management efforts can be successful.

## Future research

When it is considered that 85% of suicides occur in LMICs with largely a young population, it becomes imperative to gain a better understanding of the sociocultural factors that drive suicidality and associated comorbidities such as depression. The link between hopelessness and suicide is well-established, especially as a strong predictor of suicide, however most of the research on hopelessness has focused on adult populations. While studies have pointed to the value of early detection of hopelessness among adolescents, there is a dearth of studies from LMICs, including African countries.

Future studies on hopelessness and suicide among children and adolescents in LMICs will need to take cognizance of certain challenges such as stigma, strong discrimination against mental health problems, made worse by over-spiritualization of health problems, as well as the criminalization of suicide attempt by law in some LMICs.

Qualitative research will be vital to deepen the understanding of the meaning (s) of hopelessness and the context in which these high prevalence rates for suicide obtain so as to guide culturally appropriate suicide prevention and management efforts. We take cognizant of the fact that the factors associated with suicide in low and middle income countries are similar to those in the developed world. However, the interventions have to be tailored to the local settings.

## Author contributions

GR wrote the first draft of this editorial. All authors were guest editors for the articles submitted in response to the topic on hopelessness and suicide among children and adolescents in low and middle income countries. All authors reviewed the editorial and approved its submission for publication.
